# Maternal and child nutrition must be at the heart of the climate change agendas

**DOI:** 10.1111/mcn.13444

**Published:** 2022-10-19

**Authors:** Rafael Pérez‐Escamilla, Victoria Hall Moran

**Affiliations:** ^1^ Yale School of Public Health New Haven CT 06510 USA; ^2^ Maternal and Child Nutrition co‐Editors‐In‐Chief; ^3^ Centre for Global Development University of Central Lancashire Preston PR1 2HE UK

Climate change‐induced disasters are increasing in intensity across the world. The resulting extreme events such as hurricanes, tornadoes, floodings, sustained droughts and forest fires are already having a strong negative impact on public health because of famine, food insecurity, malnutrition, gastrointestinal and respiratory infections, air and water pollution, increased mortality rates, and exacerbated poverty resulting from disruptions in livelihoods (Cappelli et al., 2021). As a result, climate change emergency response and long‐term mitigation has become extremely urgent. United Nations (UN) agencies and many governments are developing agendas for strategies to prevent further the diverse climate‐change induced public health catastrophes that are causing massive internal displacement and cross border migrations. The vast majority of those forced from their homes because of climate change live in developing countries, and women and children are amongst the most vulnerable to its consequences, with displaced women facing increased risk of gender‐based violence, including domestic violence, forced marriage and trafficking (CARE International, 2020). UN and government agendas, however, are not paying enough attention to the disproportionate impact that climate change has on the health and food and nutrition security of women, infants, and children living in low‐income settings (Blakstad & Smith, 2020; CARE International, 2020; Pope et al., 2021).

Women, infants, and children are strongly vulnerable to climate change for several reasons. Climate change events create conditions that make it difficult for infants to breastfeed in both low‐ and high‐ income settings (Grubesic & Durbin, 2022; Zadkovic; Lombardo, and Cole, 2021). Given how crucial breastfeeding is for child health and development it is imperative that it is protected and supported during climate‐related events (Cappelli et al., 2021; Zadkovic; Lombardo, and Cole, 2021). Maternal, fetal, neonatal and child health are being disproportionately affected from the increasingly frequent outbreaks of infectious diseases related to climate change (Blakstad & Smith, 2020). Indeed, alterations to environmental conditions related to climate are enhancing the transmission potential of diverse water‐, air‐, food‐ and vector‐borne pathogens, which in turn increases even more the burden of malnutrition (Fanzo & Downs, 2021) especially among women, infants, and children. Climate change‐induced food insecurity and famines prevent women, infants, and children from meeting their nutritional needs and receiving the primary health care needed, putting at risk their health and development. If pregnant women do not meet their nutritional needs, then their health and wellbeing is negatively affected together with having an increased risk of delivering low birthweight babies (Blakstad & Smith, 2020) and long‐term adverse health outcomes for their offspring. Water insecurity resulting from frequent and severe droughts and the breakdown of sanitary infrastructure is strongly related to food insecurity affecting women and children in a disproportionate way (Blakstad & Smith, 2020; CARE International, 2020). In many communities, climate change is pushing husbands or male partners to search for income generation activities away from where their families are located, leaving women with additional caregiving and provision responsibilities than before the climate change related crisis happened, in the context of unfathomable livelihood challenges (Blakstad & Smith, 2020). Prevailing gender inequalities in low‐ and middle‐ income countries often intersect with other vulnerabilities that limit women and girls' decision‐making power, hindering their access to resources and basic services and their ability to manage and recover from the impacts of climate‐related disasters (CARE International, 2020).

To prevent further climate change shocks and their disproportionate impact on the wellbeing of women and children, it is crucial to transform the current failing food systems into healthy and sustainable food systems (Fanzo & Downs, 2021) that support breastfeeding and access to affordable healthy foods and beverages preconceptionally and during pregnancy, lactation, and early childhood (Pope et al., 2021). The current food systems that are loaded with ultra‐processed foods and sugar sweetened beverages are not only unhealthy for people but also for the planet as they are major contributors to greenhouse gases, water depletion and environmental degradation globally (Fanzo & Downs, 2021; Swinburn et al., 2021; Willett et al., 2019). Moving forward it is key that breastfeeding is supported during climate change related humanitarian emergencies (Grubesic & Durbin, 2022) as well as during normal circumstances. This is because breastfeeding improvements can help mitigate climate change as it is a “green” infant feeding practice compared to infant formula (Pope et al., 2021; Smith, 2019). Given how crucial dietary diversity is for healthy eating across the life course, the new generation of healthy and sustainable food systems must be built taking into account sustainable and equitable agriculture practices (Fanzo & Downs, 2021) that prioritize biodiversity.

Climate change repercussions have arrived and are here to stay. Strategies to mitigate its consequences need to place front and center the need to protect the food security and nutrition of women, infants, and young children. An equity lens is needed as climate change is exacerbating existing inequities by having a disproportionate impact in lower income countries (responsible for less than 4% of climate change‐causing greenhouse gases; CARE International, 2020), and among women and the poorest population segments within countries (Blakstad & Smith, 2020; Cappelli et al., 2021). Moving forward it must be acknowledged that the current Sustainable Development Goals do not acknowledge the increased risk that climate change represents for maternal, newborn, and child health and nutrition, and that there is a need to address these risks through equitable decisive actions. It is our hope that the food security and wellbeing of women, children, and their families in the context of climate change gets strongly prioritized at the upcoming 27^th^ Conference of the Parties to the United Nations Framework Convention on Climate Change (COP27) in Egypt.

## CONFLICT OF INTEREST

The authors did not receive any funding for writing this editorial and do not have any conflict of interest to declare.
